# The size and composition of haplotype reference panels impact the accuracy of imputation from low-pass sequencing in cattle

**DOI:** 10.1186/s12711-023-00809-y

**Published:** 2023-05-11

**Authors:** Audald Lloret-Villas, Hubert Pausch, Alexander S. Leonard

**Affiliations:** Animal Genomics, ETH Zürich, Universitätstrasse 2, Zürich, 8092 Switzerland

## Abstract

**Background:**

Low-pass sequencing followed by sequence variant genotype imputation is an alternative to the routine microarray-based genotyping in cattle. However, the impact of haplotype reference panels and their interplay with the coverage of low-pass whole-genome sequencing data have not been sufficiently explored in typical livestock settings where only a small number of reference samples is available.

**Methods:**

Sequence variant genotyping accuracy was compared between two variant callers, GATK and DeepVariant, in 50 Brown Swiss cattle with sequencing coverages ranging from 4- to 63-fold. Haplotype reference panels of varying sizes and composition were built with DeepVariant based on 501 individuals from nine breeds. High-coverage sequence data for 24 Brown Swiss cattle were downsampled to between 0.01- and 4-fold to mimic low-pass sequencing. GLIMPSE was used to infer sequence variant genotypes from the low-pass sequencing data using different haplotype reference panels. The accuracy of the sequence variant genotypes that were inferred from low-pass sequencing data was compared with sequence variant genotypes called from high-coverage data.

**Results:**

DeepVariant was used to establish bovine haplotype reference panels because it outperformed GATK in all evaluations. Within-breed haplotype reference panels were more accurate and efficient to impute sequence variant genotypes from low-pass sequencing than equally-sized multibreed haplotype reference panels for all target sample coverages and allele frequencies. F1 scores greater than 0.9, which indicate high harmonic means of recall and precision of called genotypes, were achieved with 0.25-fold sequencing coverage when large breed-specific haplotype reference panels (n = 150) were used. In absence of such large within-breed haplotype panels, variant genotyping accuracy from low-pass sequencing could be increased either by adding non-related samples to the haplotype reference panel or by increasing the coverage of the low-pass sequencing data. Sequence variant genotyping from low-pass sequencing was substantially less accurate when the reference panel lacked individuals from the target breed.

**Conclusions:**

Variant genotyping is more accurate with DeepVariant than GATK. DeepVariant is therefore suitable to establish bovine haplotype reference panels. Medium-sized breed-specific haplotype reference panels and large multibreed haplotype reference panels enable accurate imputation of low-pass sequencing data in a typical cattle breed.

**Supplementary Information:**

The online version contains supplementary material available at 10.1186/s12711-023-00809-y.

## Background

More than one million cattle are genotyped every year using the microarray technology for the purpose of genomic prediction [1]. Access to whole-genome sequence variants can improve the accuracy of genomic predictions and facilitates the monitoring of trait-associated alleles [2]. However, costs are still too high to sequence all individuals from a population to a sufficient coverage for calling variants.

Low-coverage whole-genome sequencing (lcWGS) followed by genotype imputation has emerged as an alternative with comparable costs to genotyping microarrays but with substantially higher marker density (tens of millions versus tens of thousands) to obtain genotypes for a target population [3–6]. Sequencing coverage as low as 0.1-fold can be used to infer sequence variant genotypes that are as accurate as those obtained from genotyping microarrays, especially for rare variants, while sequencing coverage greater than 1-fold can have much higher accuracy [5]. For many imputation methods, reference panels that are representative for the target populations are a prerequisite for the accurate imputation of genotypes from lcWGS [7–9]. The 1000 Genomes Project (1KGP) and the Haplotype Reference Consortium (HRC) established such reference panels for several human ancestry populations [10, 11] and made them available through dedicated imputation servers [12]. A bovine imputation reference panel established by the 1000 Bull Genomes project is frequently used to infer sequence variant genotypes for large cohorts of genotyped taurine cattle, thus enabling powerful genome-wide analyses at the nucleotide level [13]. Sequenced reference panels are available for other animal species [14, 15], but they lack diversity as they were established mainly using data from mainstream breeds and thus are depleted for individuals from local or rare populations.

An exhaustive set of variants and accurate genotypes are crucial to compile informative haplotype reference panels. The Genome Analysis Toolkit (GATK) has been frequently applied to discover and genotype sequence variants in large reference populations of many livestock species [3, 14]. DeepVariant has recently emerged as an alternative machine learning-based variant caller [16]. Several studies suggest that DeepVariant has superior genotyping accuracy compared to GATK [17–20]. However, DeepVariant has rarely been applied to call variants in species other than humans [21, 22].

In this study, we benchmark sequence variant genotyping of DeepVariant and GATK in a livestock population. Then, we build haplotype reference panels of varying sizes and composition with DeepVariant, and use GLIMPSE to impute sequence variant genotypes for cattle that had been sequenced at between 0.01- and 4-fold. We show that within-breed haplotype reference panels outperform multibreed reference panels across all tested scenarios, provided that a sufficient number of sequenced samples is available.

## Methods

### Data availability and code reproducibility

Short paired-end whole-genome sequencing reads from 501 cattle from nine breeds were used: 327 Brown Swiss (BSW), 50 Fleckvieh, 13 Hereford, 57 Holstein, 2 Nordic Red, 14 Rätisches Grauvieh, 10 Simmental, 25 Tyrolean Grauvieh and 3 Wagyu cattle. Accession numbers for the raw data are available in Additional file 1.

Computational workflows were implemented using Snakemake [23] (version 7.5.0 or newer). The R software environment (version 4.0.2) and ggplot2 package [24] (version 3.3.2) were used to create figures and perform statistical analyses.

Scripts and workflows are available online:

https://github.com/AnimalGenomicsETH/Low_pass_imputation

### Alignment, mapping quality and depth of coverage

Raw short sequencing reads were filtered with fastp [25] (version 0.23.1), and MultiQC [26] (version 1.11) was applied to collect the quality metrics across samples. Reads were split per read groups with gdc-fastq-splitter [27] (version 1.0.) and subsequently aligned with bwa-mem2 [28] using the *-M* and *-R* flags to a manually curated version of the current bovine Hereford-based reference genome (ARS-UCD1.2) [29] that included a Y chromosome as described in [30].

Samblaster [31] (version 0.1.26), Sambamba [32], samtools [33, 34] (version 1.12), and Picard tools [35] (version 2.25.7) were used to deduplicate and sort the BAM files.

We calculated average coverage with mosdepth [36] (version 0.3.2) considering all aligned reads that had a mapping quality (MQ) $\ge$ 10.

### Comparison between variant callers

#### Testing set

Fifty BSW cattle with coverages ranging from 4 to 63-fold were selected as testing set for a comparison between GATK and DeepVariant.

#### GATK

We used the BaseRecalibrator module of GATK [37, 38] (version 4.2.2.0) to adjust the base quality scores of the deduplicated bam files using 115,815,224 unique positions from the Bovine dbSNP version 150 as known variants. Multi-sample variant calling was performed with the GATK HaplotypeCaller, GenomicsDBImport and GenotypeGVCFs modules according to the best practice guidelines [39, 40]. We applied the VariantFiltration module for site-level filtration using the thresholds indicated in [30] to retain high-quality single nucleotide polymorphisms (SNPs) and insertion/deletion variants (INDELs).

#### DeepVariant + GLnexus

DeepVariant [16] (version 1.2) was run on the deduplicated bam files using the *WGS* Illumina-trained model, producing a gVCF output per sample. The gVCF files were then merged and filtered using GLnexus [41] (version 1.4.1) with the *DeepVariantWGS* configuration but with the *revise_genotypes* flag set to false.

#### VCF imputation and statistics

We used Beagle 4.1 [42] (27Jan18.7e1) to improve genotype calls and impute sporadically missing genotypes from genotype likelihoods (*gl* mode). INDELs were left-normalised using bcftools [34] (version 1.12 or 1.15) *norm*. Variant and genotype counts, and Ti:Tv ratios were calculated with bcftools *stats* and bcftools *query*. VCF files were indexed with tabix [43, 44].

#### Variant annotation

Functional consequences of SNPs were predicted based on the Ensembl (release 104) annotation of the bovine reference assembly using the Variant Effect Predictor tool (VEP) [45] (version 106) with default parameter settings.

#### Evaluation of the accuracy of variant calling

Microarray-derived genotypes from 33 cattle that also had sequence-derived genotypes (see Additional file 1) were our truth chip set. We intersected the truth (microarray) and query (WGS variants) VCF files using bcftools *isec* with both the *-c none* (exact—only matching REF:ALT alleles are allowed) and *-c all* (position—all coordinate matches are allowed) flags, and retained biallelic SNPs with bcftools *view* to compare the genotypes. Three-way intersection overlaps were counted with bedtools *multiinter* [46] and visualised with UpSetR [47, 48]. Since the microarray data contains fewer sites than WGS, we intersected the truth and query sets. Only positions where the truth genotypes were not homozygous for the reference allele (i.e., the variants that segregate within the target samples) were retained. We calculated recall (percentage of true positives in the query set), precision (proportion of matching genotypes in both truth and query sets), and F1 scores (harmonic mean of precision and recall) using hap.py [49] (version 0.3.9) on a per-sample basis. Agreement between the imputed variant alleles/genotypes and raw sequencing reads was assessed with Merfin’s k-mer-based filtering method [50] (commit fc4f89a). A k-mer database was prepared using Meryl (commit 51fad4b) with a k-mer size of 21 and minimum k-mer occurrence of 2 in the short sequencing reads. Variants that were poorly supported, i.e., the alternate sequence (variant and flanking regions) appeared less often in k-mers than the reference sequence did in a genotype-aware proportion, were filtered out.

We assessed Mendelian consistency in filtered but not-imputed data from parent-offspring pairs and trios (see Additional file 2) using the bcftools *+mendelian* plugin [34]. We calculated discrepancy rate as the number of inconsistent sites divided by the total number of non-missing sites. For duos (dam-offspring or sire-offspring) only homozygous sites were considered. Assessing discrepancy was only possible when the parent genotype was homozygous (0/0 or 1/1).

### Imputation of low-pass sequencing data

#### Generation of the haplotype panels

The BSW reference panels contained 150, 75 and 30 samples that were randomly selected from 303 BSW samples. The non-BSW panels contained 150, 75 and 30 samples that were randomly selected from 174 non-BSW samples. The multibreed panels were randomly selected from a combination of the above, and they contained 150 samples of which 50%, 25%, and 10% were BSW samples and the remaining were non-BSW. Three random replicates for each panel were created. Sequence variant genotypes were called for each panel with DeepVariant and sporadically missing genotypes were imputed with Beagle 4.1 [42] (27Jan18.7e1) as described above.

#### Truth sequencing set, truth variants and subsampling

Variants were called with DeepVariant and GLnexus as described previously for 24 BSW samples with a coverage higher than 20-fold to generate a truth set for assessing imputation accuracy. The raw whole-genome sequencing reads of the 24 BSW samples were then downsampled with seqtk [51] to mimic 4x, 2x, 1x, 0.5x, 0.25x, 0.1x, and 0.01x coverage, and subsequently aligned to ARS-UCD12 as described previously.

Genotype likelihoods for the variants that are present in the haplotype reference panel were estimated from the subsampled read alignments with bcftools *mpileup* and bcftools *call*. These were then imputed using the different haplotype panels and GLIMPSE [52] (version 1.1.1). We used 2-Mb windows and 200-kb buffer sizes during the chunk step followed by phasing and ligation to produce the final imputed variant calls.

#### Comparison of true and imputed variants

The accuracy of the imputed sequence variant genotypes was assessed with hap.py as described above. The minor allele frequency (MAF) of the imputed sequence variants was calculated with PLINK [53] (version 1.9). The estimated imputation quality was retrieved from the INFO flag from the VCF files produced by GLIMPSE with bcftools *query*. Pearson squared correlation between expected and actual dosages ($r^{2}$) was calculated with the bcftools *stats*.

**Fig. 1:**
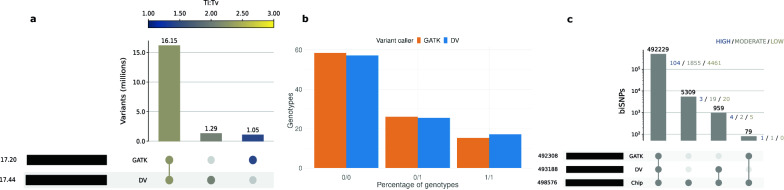
Comparison of the variants called between DeepVariant (DV) and GATK. **a** Intersection of variants called with each variant caller (or both) and the Ti:Tv ratio of the biallelic SNPs of each set. **b** Percentage of imputed genotypes called by each variant caller. **c** Intersection of variant calls with truth genotyping arrays, where only variants at intersecting positions are retained. Variants with a low, moderate and high predicted impact from the intersecting sets are indicated

**Table 1:** Summary of the variants called by GATK and DeepVariant (DV)

Variant caller	Sets	Variants	SNPs	INDELs	Ti:Tv ratio	High impact predicted SNPs / INDELs
GATK	Raw	18,654,649 (831,391)	16,135,130 (58,049)	2,617,546 (773,342)	2.16	2680 / 4493
GATK	Filtered-out	1,453,366 (239,008)	1,271,522 (8577)	279,871 (230,431)	1.66	428 / 500
GATK	Filtered	17,201,283 (592,383)	14,863,608 (49,472)	2,337,675 (542,911)	2.20	2252 / 3993
DV	Raw	18,748,114 (702,173)	16,554,438 (54,438)	2,401,933 (647,735)	2.24	3530 / 2778
DV	Filtered-out	1,571,454 (270,963)	1,174,815 (11,834)	393,927 (259,108)	2.19	1061 / 612
DV	Filtered	17,440,238 (577,997)	15,361,785 (42,899)	2,240,627 (535,098)	2.24	2474 / 2240

Multiallelic sites are presented in parentheses. Ti:Tv ratios are restricted to biallelic SNPs. Functional consequences are predicted for biallelic SNPs / biallelic INDELs

## Results

### Variant calling with GATK and DeepVariant

We compared sequence variant calling between GATK and DeepVariant for 50 Brown Swiss (BSW) cattle for which the sequencing depth ranged from 4 to 63-fold (19.26 ± 11.09) along the autosomes. GATK and DeepVariant identified 18,654,649 and 18,748,114 variants, respectively, of which 7.79% and 8.38% were filtered out because of their low quality (Table 1). In total, 16,147,567 filtered variants were identified by both callers, but 1,053,716 and 1,292,671 variants were private to GATK and DeepVariant, respectively (Fig. 1a). Overall, DeepVariant had more private SNPs than GATK, but GATK had more private INDELs than DeepVariant (see Additional file 3: Table S1). 416,642 variants had the same coordinates but different alternative alleles. These discrepant sites were primarily INDELs (83%, as opposed to the 12% of INDELs in all shared variants). Multiallelic sites accounted for 3.44% and 3.31% of the variants (0.33% and 0.28% of the SNPs, and 23.22% and 23.94% of the INDELs) that passed the quality filters of GATK and DeepVariant, respectively. Multiallelic sites were enriched among the variants private to either GATK or DeepVariant (see Additional file 3: Table S2).

The biallelic variants called by GATK had a higher percentage of homozygous reference (HOMREF) and heterozygous (HET) genotypes whereas the biallelic variants called by DeepVariant had a higher percentage of homozygous alternative (HOMALT) genotypes (Fig. 1b and see Additional file 4: Fig. S1a). Missing genotypes were very rare (<0.01%) for GATK-called biallelic variants but accounted for 2.72% of the DeepVariant-called genotypes (see Additional file 4: Fig. S1b). Beagle phasing and imputation increased the number of HET genotypes for both GATK (mostly transitioning from HOMREF) and DeepVariant (mainly due to the refinement of missing genotypes) (see Additional file 4: Fig. S1c).

Functional consequences on the protein sequence were predicted for all biallelic variants. DeepVariant identified 9% more SNPs that were predicted to have a high impact on protein function than GATK (Table 1 and see Additional file 3: Table S3). Around one fourth of the high impact SNPs detected by DeepVariant (24%) were not detected by GATK. GATK identified 78% more INDELs that were predicted to have a high impact on protein function than DeepVariant. More than half of the high impact INDELs detected by GATK (52%) were not detected by DeepVariant.

We investigated the ratio of transitions to transversions (Ti:Tv) to assess variant quality. Deviations from an expected genome-wide Ti:Tv ratio of $\sim$ 2.0$-$2.2 indicate random genotyping errors or sequencing artifacts [17, 20, 38, 54]. The Ti:Tv ratio was 2.16 and 2.24 for raw SNPs identified by GATK and DeepVariant, respectively (Table 1). While the Ti:Tv ratio was higher (2.20) for the GATK variants that met the quality filters, variant filtration had no impact on the Ti:Tv ratio for SNPs called by DeepVariant. The Ti:Tv ratio of the filtered-out SNPs was substantially lower for GATK (1.66) than for DeepVariant (2.19). SNPs private to GATK had lower Ti:Tv ratios than those private to DeepVariant (Fig. 1a). Substantial differences in the Ti:Tv ratio (0.81 points) were observed between overlapping and GATK-private SNPs but were smaller (0.18 points) between overlapping and DeepVariant-private SNPs.

### Accuracy of variant calling

Thirty-three sequenced cattle also had between 17,575 and 490,174 SNPs genotyped with microarrays. The filtered biallelic SNPs called with GATK and DeepVariant (query sets) were compared to those genotyped with the microarrays (truth chip set). The vast majority (98.82%) of the SNPs present in the truth chip set was called by both tools (Fig. 1c). The number of overlapping SNPs present in the truth chip set was slightly larger for DeepVariant than for GATK. 1.06% (n = 5309) of the SNPs present in the truth chip set were not called by any of the software as biallelic SNPs. However, 3497 of these SNPs were present at the same position but had different alternative alleles (e.g., multiallelic SNPs or INDELs) in DeepVariant versus GATK while the other 1812 positions were truly missing. Most of the biallelic SNPs private to the chip set (5265) were also missing in the raw calls from the variant callers. DeepVariant filtered out more variants present in the truth chip set than GATK.

The analysis of variant effect predictions for the filtered variants revealed that most low/moderate/high impact variants were called by both GATK and DeepVariant (99.4%, 98.8%, and 92.8%, respectively). However, DeepVariant additionally called 5/2/4 biallelic SNPs predicted as low/moderate/high impact respectively, while GATK only called 0/1/1 (Fig. 1c). Some of the low/moderate/high impact biallelic SNPs private to GATK (1 out of the 2) and DeepVariant (5 out of the 11) were called either as multiallelic SNPs or as INDELs by the other caller (see Additional file 3: Table S4). Only half (1 out of 2) of GATK’s private variants have a MAF higher than 0.5, while most (9 out of 11) of the DeepVariant’s private variants do, which suggests that GATK misses more variants that might have a larger impact in populations.

### Genotyping accuracy of variant calls

GATK and DeepVariant called 492,265 and 493,145 variants from the truth chip set, respectively. GATK missed (8.13%) and miscalled (10.13%) more truth variants than DeepVariant. Around 90.6% of the discrepancies between the sequence variant genotypes and the truth chip set in both variant callers were due to missing genotypes in the sequence set. Of those, GATK missed proportionally more HOMALT than DeepVariant, and DeepVariant missed proportionally more HET variants. For the remaining $\sim$ 9.4% of mismatching genotypes (miscalled), GATK miscalled proportionally more HOM variants, and DeepVariant significantly miscalled proportionally more HET variants (see Additional file 4: Fig. S2). However, after imputation, the proportion of HET positions miscalled was higher in the GATK set and the proportion of HOMREF positions miscalled as HET was significantly higher in the DeepVariant set.

**Fig. 2:**
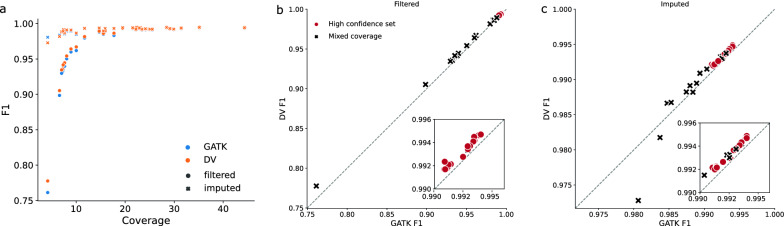
Comparison of the F1 values obtained with hap.py from GATK and DeepVariant (DV) variant calls against the truth chip set for 33 samples. **a** Imputation improves genotype accuracy for sequence coverages lower than 20x but has little impact for sequence coverages higher than 20x. **b** DV has a higher F1 score for every sample than GATK for post-filter variants. The high confidence set indicates the 17 microarray genotyped samples out of the 24 samples used later as a truth set for GLIMPSE imputation. **c** Similar to (**b**) but for post-imputation variants

Recall, precision and F1 score of the filtered query sets were calculated to assess the genotyping accuracy for both variant callers. DeepVariant had strictly better F1 scores than GATK for the filtered data (mean of 0.9719 versus 0.9694, Fig. 2a and b). The difference was small but significant (Wilcoxon signed-rank test, p=$2.3\text {x}10^{-10}$). As expected, lower coverage (<20x) samples benefited from imputation, improving their F1 scores to values that were comparable to high-coverage samples. Imputation improved GATK genotypes more than DeepVariant genotypes at lower coverages, which could be due to better calibration of genotype likelihoods, but DeepVariant was still strictly better for coverage-folds higher than 7x. Overall, DeepVariant still had a significantly higher mean F1 score for the imputed data (0.9912 versus 0.9907, Wilcoxon signed-rank test p=$4.2\text {x}10^{-05}$, Fig. 2c).

**Fig. 3:**
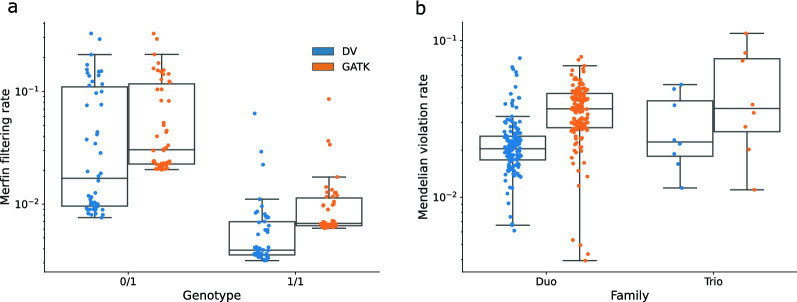
Genotyping accuracy of variant calls validated with sequencing reads and Mendelian relationships. **a** Filtering rate of heterozygous (0/1) and homozygous alternate (1/1) variant calls post-imputation for GATK and DV. Higher filtering rate indicates the genotype/allele is not consistent with k-mers from the same-sample sequencing reads. **b** Mendelian violation rate for 206 separate samples, with either 2 family members (Duo) or all 3 (Trio). Mendelian violations are defined as genotypes in the offspring that could not have been inherited from the parents. In the case of duos, only variants homozygous in the parent can be confirmed as violations of Mendelian inheritance

We examined variant genotyping accuracy through Merfin [50]. Merfin filters out variants when the proportion of “reference” and “alternate” k-mers for that variant from the sample’s short sequencing reads does not match the genotype and thus is likely incorrect. HET genotypes obtained with both GATK and DeepVariant had less support from the sequencing reads, as they are more difficult to genotype correctly than HOM genotypes. For both HET and HOMALT, more of the genotypes of DeepVariant than of GATK were supported (Fig. 3a). The difference between the tools was statistically significant for both genotypes (two-sided paired Wilcoxon test, p_HET_=$3.6\text {x}10^{-19}$, p_HOMALT_=$1.8\text {x}10^{-19}$).

In addition, we compared Mendelian concordance rate between the sequenced duos and trios across the two variant callers. There were only two family relationships in the previously examined 50 samples, and so we evaluated the concordance on a separate set of 206 samples (see Additional file 2) forming seven trios (both parents available) and 142 duos (one parent available). DeepVariant had less genotypes that are in conflict with Mendelian inheritance compared to GATK (2.3% versus 3.8%, Fig. 3b, one-sided paired Wilcoxon signed-rank test p=$1.3\text {x}10^{-24}$). This was due to DeepVariant calling both more genotypes that were compatible as well as fewer that were incompatible with parent-offspring relationship.

### Generation of a sequencing validation set for lcWGS imputation

We benchmarked the accuracy of low-pass sequence variant imputation in a target population consisting of 24 BSW samples with a mean autosomal coverage of 28.12 ± 9.07-fold. DeepVariant identified 15,948,663 variants (87.77% SNPs and 12.23% INDELs) in this 24-sample cohort of which we considered 13,854,932 biallelic SNPs as truth set.

The sequencing reads of these 24 samples were randomly downsampled to mimic medium (4x and 2x), low (1x, 0.5x, 0.25x, and 0.1x), and ultralow (0.01x) sequencing coverage. We then aligned the reads to the reference sequence and produced genotype likelihoods from the pileup files. Subsequently, genotypes were imputed with GLIMPSE considering nine haplotype reference panels, and compared to the truth set to determine the accuracy of imputation.

The nine haplotype reference panels varied in size and composition. Five haplotype reference panels contained 150 cattle (full panels) of which either 0%, 10%, 25%, 50% or 100% were from the BSW breed (i.e., the breed of the target samples). The other four panels contained either 75 or 30 cattle (reduced panels) that were either from the BSW breed or from breeds other than BSW. DeepVariant identified between 17,035,514 and 28,755,400 sequence variants in the nine haplotype reference panels (Table 2). The full BSW panel contained 5,167,875 fewer biallelic SNPs than the full non-BSW panel. The 50% multibreed panel had the largest number of variants shared with the truth set and the smallest number of variants present in the truth set but missing in the reference panel, closely followed by the BSW panel. The reduced non-BSW panel (30 samples) had the smallest number of shared variants and the largest number of variants that were present in the truth but missing in the reference set.

**Fig. 4:**
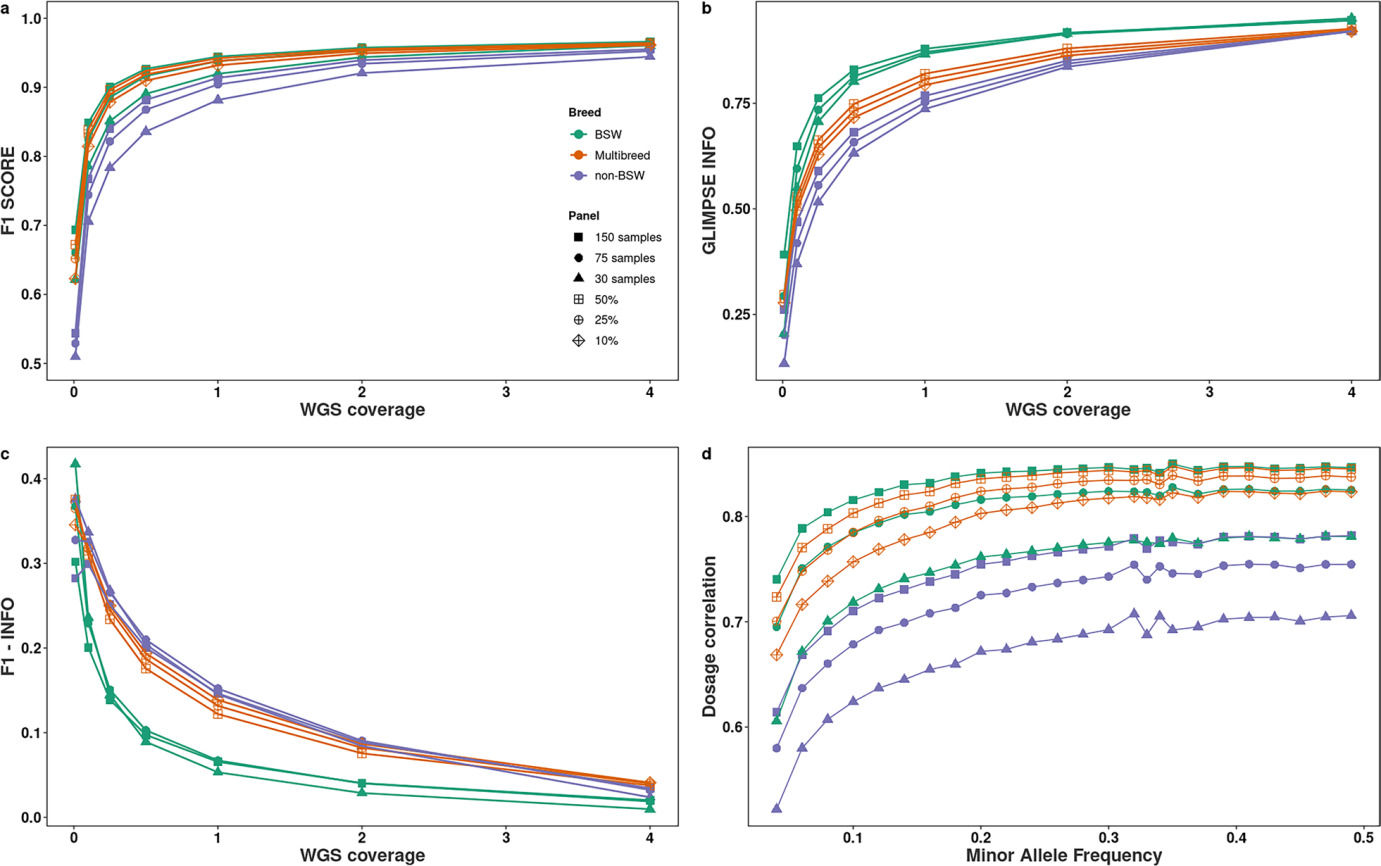
Genotyping accuracy from low-pass whole-genome sequencing. **a** F1 score between truth and imputed variants. **b** GLIMPSE INFO score achieved with different sequencing coverages and haplotype panels. **c** Differences (subtraction) between F1 and GLIMPSE INFO average scores for different sequencing coverages and haplotype panels. **d** Squared dosage correlation (r$^{2}$) between imputed data and truth set, stratified by MAF for lcWGS at 0.5x. Panels are indicated with colours and number/percentages of BSW samples are indicated with different shapes of points. Multibreed panels contain 150 samples. Points indicate the average of the results for all variants in three replicates

**Table 2:** General overview of the haplotype reference panels: number of samples, coverage and number of variants called

Panel	Samples	Coverage	Variants	Biallelic SNPs	SNPs shared truth-query sets	Truth SNPs missing in haplotype panel	SNPs private to haplotype panel
BSW	150	9.40	22,493,568	19,682,362	13,537,126	317,806	6,145,236
BSW	75	9.65	19,883,488	17,345,201	13,373,462	481,470	3,971,739
BSW	30	9.42	17,035,514	14,839,600	12,810,541	1,044,391	2,029,059
Multibreed (50%)	150	10.48	27,710,504	24,325,185	13,568,744	286,188	10,756,441
Multibreed (25%)	150	10.86	28,755,400	25,266,484	13,531,721	323,211	11,734,763
Multibreed (10%)	150	11.44	28,608,506	25,126,433	13,427,451	427,481	11,698,982
Non-BSW	150	11.78	28,303,738	24,850,237	13,075,827	779,105	11,774,410
Non-BSW	75	11.78	25,059,239	21,968,792	12,868,909	986,023	9,099,883
Non-BSW	30	11.45	21,011,311	18,402,870	12,283,284	1,571,648	6,119,586

Shared and private variants are considered through exact matching (position and alleles). Values are the mean of 3 replicas per haplotype panel

### Assessment of lcWGS imputation with the different haplotype panels

Increasing the number of reference haplotypes enabled higher F1, recall and precision scores in all tested scenarios (Fig. 4a and see Additional file 3: Table S5). Imputation accuracy also improved with increasing lcWGS coverage, with the biggest change between 0.01x and 1x coverage, and continued to improve with diminishing returns between 1x and 4x coverage. The difference in accuracy between panels also decreased as coverage increased.

The largest BSW haplotype reference panel (n = 150) performed better than any of the multibreed panels at all sequencing coverages. Multibreed panels outperformed BSW panels with a larger number of BSW samples, especially at low coverage. For instance, a large multibreed panel containing 10% BSW samples (n = 15) produced higher F1 scores than a smaller breed-specific panel containing two times more BSW samples (n = 30). Similarly, a large multibreed panel containing 25% BSW samples (n = 37) provided higher F1 scores than a smaller breed-specific panel containing two times more BSW samples (n = 75) for lcWGS below 1-fold coverage. Accuracies were similar between large multibreed panels and smaller breed-specific panels when the coverage of the lcWGS was higher than 1-fold. All results were validated by three different conformations of the haplotype reference panels (replicas). Standard errors accounting for all the replicas did not overlap for any of the haplotype panels (see Additional file 4: Fig. S3a).

The INFO score [55] was higher for all BSW panels than for the multibreed panels across all coverages (Fig. 4b). A higher proportion of variants were imputed with an INFO score greater than 0.6 in the BSW than in non-BSW or multibreed panels (see Additional file 4: Fig. S3b). Therefore, panels for which the average INFO score was higher had also a major proportion of variants with high imputation quality, potentially selected for downstream analyses. The differences between BSW panels and the others were larger than those between multibreed and non-BSW panels. The average values of F1 and the average INFO scores were closer for the variants imputed with BSW panels (Fig. 4c). The differences between both metrics decreased as the coverage of the lcWGS increased (see Additional file 4: Fig. S3c and d).

The variants were then stratified by MAF, and the squared correlation of genotype dosages (r$^{2}$) was calculated (Fig. 4d). The correlations increased along with the MAF similarly for all the panels. The highest correlations were for BSW panel (150 samples) and multibreed panels (50% and 25%). The values increased substantially between 0$-$0.1 MAF and continued to increase slowly until the MAF reached 0.5 for all panels.

## Discussion

Higher F1 scores against a microarray truth set, improved k-mer based variant filtering, and the fewer Mendelian errors suggest that DeepVariant is a superior variant caller to GATK for bovine short read sequencing. These results extend the evidence of the DeepVariant’s greater accuracy that was established in multiple human studies [17–20]. Ti:Tv ratios in the expected range of 2$-$2.2 [38, 54] suggest that variant calls private to DeepVariant contain genuine variants, whereas the lower Ti:Tv ratio in variants private to GATK indicate an excess of false positives. DeepVariant revealed more SNPs that have an impact based on their annotation, likely providing additional putative trait-associated candidates for downstream analyses. DeepVariant was approximately 3.5x faster in end-to-end variant calling compared to GATK, due to greater multithreading potential and to the fact that it does not require pre-processing like GATK’s base recalibration step (see Additional file 3: Table S6). The peak memory usage was approximately 65% higher for DeepVariant than for GATK (81 GB versus 49 GB). Although our work focused on CPU-only machines, DeepVariant also offers GPU acceleration (roughly 1.9x faster overall), while GATK has no official GPU support, although there are third-party developments (roughly 1.4x faster overall) [56].

To the best of our knowledge, our study is the first to establish bovine haplotype reference panels with DeepVariant. A within-breed panel consisting of 75 samples enabled us to genotype more than 13 million sequence variants in animals sequenced at a 0.5-fold sequencing coverage with F1 scores greater than 0.9. Larger haplotype reference panels (n = 150) from the same breed as the lcWGS data outperform multibreed panels across the whole low coverage spectrum (from 0.1- to 1-fold) and MAF, including rare variants. The development of such panels is a feasible alternative to using much larger multibreed panels, such as the 1000 Bull Genomes project imputation reference panel [13]. Such large panels, encompassing huge within- and across-breed diversity, may be regarded as the most complete and thus best genomic resources available in bovine genomics. However, using such large panels may be detrimental for breed-specific imputation (also described by Nawaz et al. [57]), as we observed many relevant sites were filtered out before imputation due to being multiallelic, resulting in a lower F1 score than the 75 sample BSW panel at 1-fold coverage and higher. The use of within-breed panels is also more computationally efficient and are 18 to 33% faster than that of multi- or different-breed panels of the same size (see Additional file 4: Fig. S4), and approximately 7 times faster than using the 1000 Bull Genomes Project panel.

In absence of an adequately sized breed-specific panel (e.g., less than 30 animals), F1 scores of 0.9 can also be reached either by increasing the coverage of the lcWGS or by adding distantly related samples from other breeds to the haplotype panels as even animals from seemingly unrelated breeds may share short common haplotypes. Both options will provide accurate sequence variant genotypes at affordable costs for samples from rare breeds, where large breed-specific haplotype reference panels cannot be easily established. For instance, F1 scores > 0.92 are observed at a 2-fold sequencing coverage for all tested haplotype panels with small differences among them. This is likely because higher coverages provide more information for imputation from the own sequencing reads, while lower coverages rely on the information from haplotypes in the panels. We also achieved F1 scores of 0.9 with large multibreed panels containing only 10% of within-breed samples (n = 15). However, reference panels that contain only few samples from the target breed are in general less informative as evidenced by the lack of about 100K truth SNPs that were present in same-size breed-specific panels. Additionally, a threshold of non-related haplotypes from which only marginal gains to imputation accuracy are observed have been described [15, 57, 58]. Overall results are compatible with similar studies with haplotype panels of both larger and smaller sample sizes [15, 57, 59]. Genotypes imputed from lcWGS enable the prediction of genomic breeding values and facilitate powerful genome-wide association studies at nucleotide resolution [3, 60].

Although imputation accuracy (F1) and GLIMPSE’s predicted imputation accuracy (INFO score) are respectively averaged over each sample and each variant, we note that F1 (truth) is strictly higher than INFO (estimation). The differences appear to be more pronounced for reference haplotype panels that are constituted from a different breed to the target sample and at lower coverages (i.e., less than 0.25-fold coverage, where GLIMPSE’s INFO scores are inaccurate [6]). While, for example, multibreed panels are nearly as equally accurate as the 150 sample BSW panel, the INFO scores are notably lower. Similarly, the INFO score drops more rapidly for lower coverages, suggesting that a fixed threshold may be unnecessarily conservative given the slower decay in F1. The GLIMPSE INFO score is also positively correlated with variant MAF, and thus filtering based on INFO predominantly removes low-frequency variants. While INFO and other imputation accuracy scores are still useful, additional care should be taken in determining a constant filtering threshold as more and different panels become available for use.

## Conclusions

DeepVariant outperforms GATK for calling variants from bovine short sequencing reads and can be readily used to establish informative haplotype reference panels. Medium-sized breed-specific haplotype reference panels enable accurate imputation of millions of sequence variant genotypes from low-pass (0.5-fold) sequence data. The same degree of accuracy of the imputed genotypes is achieved from larger multibreed reference panels that lack individuals from the target breed but contain individuals from distantly related breeds. Increasing the sequencing coverage compensated to a certain extent the lack of representative animals in the reference panels. Nevertheless, suboptimal haplotype reference panels lack variants private to the breed under study, especially rare variants.

## Supplementary Information

**Additional file 1.** Raw data: accession numbers and mapping file.

**Additional file 2.** Composition of parent-offspring pairs and trios for Mendelian consistency check.

**Additional file 3** **Table S1.** Percentage of overlapping variants across the different GATK and DV sets. **Table S2.** Number of total and multiallelic SNPs shared and private for the different GATK and DV sets. **Table S3.** Biallelic variantsannotated with VEP and classified depending on the likely functional effects: high, moderate, low and modifier. **Table S4.** VEP annotation of GATK and DV private variants. **Table S5.** F1, recall and precision scores when comparing the truth set and the query sets. **Table S6.** Compute resources used by DeepVariantand GATK to pre-process aligned BAM files, call variants per sample, and jointly genotype and filter variants.

**Additional file 4** **Figure S1.** Summary of genotypes. **Figure S2.** Genotyping accuracy of variant calls. **Figure S3.** Genotyping accuracy from low-pass whole-genome sequencing. **Figure S4.** CPU hours required to impute different coverages and panels for 3 replicates.

## Data Availability

Sequencing data used in this study are listed in Additional files 1 and 2. Scripts and workflows are available on Github.
