# Supplementary material for: The size and composition of haplotype reference panels impact the accuracy of imputation from low-pass sequencing in cattle
Source: Genet Sel Evol. 2023 May 11;55:33. doi: 10.1186/s12711-023-00809-y (PMC10173671; doi:10.1186/s12711-023-00809-y)
Supplement: Supplementary file 3 — Additional file 3 Table S1. Percentage of overlapping variants across the different GATK and DV sets. Table S2. Number of total and multiallelic SNPs shared and private for the different GATK and DV sets. Table S3. Biallelic variantsannotated with VEP and classified depending on the likely functional effects: high, moderate, low and modifier. Table S4. VEP annotation of GATK and DV private variants. Table S5. F1, recall and precision scores when comparing the truth set and the query sets. Table S6. Compute resources used by DeepVariantand GATK to pre-process aligned BAM files, call variants per sample, and jointly genotype and filter variants. [file 12711_2023_809_MOESM3_ESM.pdf]

Additional Tables

Table S1. *Percentage of overlapping variants across the different GATK and DV sets.*

		Overlapping Variants (%)	Overlapping SNPs (%)	Overlapping INDELs (%)
**Biallelic**	**GATK**	93.39 (93.55)	95.37 (95.38)	77.97 (79.35)
	**DV**	91.98 (92.15)	92.61 (92.71)	86.47 (87.18)
**Multiallelic**	**GATK**	37.17 (60.45)	24.73 (25.82)	41.26 (71.84)
	**DV**	38.10 (61.95)	17.52 (27.94)	34.41 (60.02)

Two different intersection modes were used: exact match (same coordinates, and REF and ALT alleles) and position match (only coordinates were queried - in parentheses).

Table S2. *Number of total and multiallelic SNPs shared and private for the different GATK and DV sets.*

	Overlapping variants			Private variants		
	**SNPs**	**Multiallelic SNPs**	**Multiallelic SNPs (%)**	**SNPs**	**Multiallelic SNPs**	**Multiallelic SNPs (%)**
**GATK**	14,071,834 (14,134,549)	36,257 (45,367)	0.26 (0.32)	791,774 (729,059)	13,215 (4,105)	1.67 (0.56)
**DV**	14,071,834 (14,244,141)	36,257 (39,134)	0.26 (0.27)	1,289,951 (1,117,644)	6,642 (3,765)	0.51 (0.34)

Two different intersection modes were used: exact match (same REF and ALT alleles) and position match (only coordinates were queried - in parentheses).

Table S3. *Biallelic variants (SNPs / INDELs) annotated with VEP and classified depending on the likely functional effects: high, moderate, low and modifier.*

	Set	High	Moderate	Low	Modifier
**GATK**	Raw	2,680 / 4,493	67,180 / 1,657	90,955 / 3,403	15,667,553 / 1,985,337
	Raw private	546 / 2,283	10,390 / 674	11,327 / 990	926,538 / 452,647
	Raw shared	2,134 / 2,210	56,790 / 983	79,628 / 2,413	14,741,015 / 1,532,690
	Filtered	2,252 / 3,993	57,675 / 1,522	82,618 / 3,126	14,574,453 / 1,883,261
	Filtered private	362 / 2,089	7,503 / 598	8,714 / 820	664,190 / 401,763
	Filtered shared	1,890 / 1,904	50,172 / 924	73,904 / 2,306	13,910,263 / 1,481,498
	Filtered-out	428 / 500	9,505 / 135	8,337 / 277	1,093,100 / 102,076
**DV**	Raw	3,530 / 2,778	69,525 / 1,162	88,839 / 2,833	16,125,916 / 1,748,769
	Raw private	1,396 / 572	12,733 / 175	9,213 / 421	1,384,901 / 216,078
	Raw shared	2,134 / 2,206	56,792 / 987	79,628 / 2,412	14,741,015 / 1,532,691

	Filtered	2,474 / 2,240	58,457 / 1,068	80,765 / 2,730	15,013,146 / 1,700,025
	Filtered private	584 / 338	8,283 / 142	6,863 / 424	1,102,883 / 218,527
	Filtered shared	1,890 / 1,902	50,174 / 926	73,902 / 2,306	13,910,263 / 1,481,498
	Filtered-out	1,061 / 612	11,214 / 135	8,226 / 348	1,142,970 / 134,643

Variants were considered before and after filtering (exact match for filtered-out). Variants were also divided depending on whether were called by both variant callers (shared) or only one (private) – position matches were accepted.

***Table S4**. VEP annotation of GATK and DV private variants.*

Variant caller	Position	Predicted impact	MAF	HOMALT samples	Reason not intersecting
GATK	9:78221981	Moderate	0.78	21	INDEL
GATK	19:46232626	High	0.02	0	-
DV	5:116965831	Low	0.09	1	-
DV	6:37374718	Low	1	33	-
DV	11:15257933	Low	0.57	10	INDEL
DV	15:1094876	Low	0.75	16	-
DV	25:20777430	Low	0.13	1	-
DV	15:49709135	Moderate	0.06	2	-
DV	18:61285359	Moderate	0.08	0	Multiallelic
DV	4:49174950	High	0.02	0	INDEL
DV	7:41125658	High	0.26	1	Multiallelic
DV	10:27971463	High	0.01	0	INDEL
DV	15:49811331	High	0.29	3	-

MAFs greater than 0.05 are bolded, and HOMALT samples refers to the number of samples with 1/1 genotypes for the variant (out of a maximum of 33 genotyped samples). Some variants were present in the other caller but as a different variant than predicted by truth. Truly missing variant calls are indicated by "-".

***Table S5**. F1, recall and precision scores when comparing the truth set and the query sets (different coverages and panels).*

**Metric**	**Panel**	**4x**	**2x**	**1x**	**0.5x**	**0.25x**	**0.1x**	**0.01x**
**F1**	**BSW (150)**	**0.9663**	**0.9575**	**0.9456**	**0.9269**	**0.9009**	**0.8490**	**0.6934**
	**BSW (75)**	0.9655	0.9543	0.9381	0.9167	0.8855	0.8245	0.6609
	**BSW (30)**	0.9608	0.9436	0.9198	0.8906	0.8513	0.7853	0.6215
	**Multibreed (50%)**	0.9642	0.9554	0.9422	0.9239	0.8966	0.8385	0.6722
	**Multibreed (25%)**	0.9629	0.9529	0.9382	0.9188	0.8901	0.8296	0.6518
	**Multibreed (10%)**	0.9614	0.9494	0.9320	0.9099	0.8786	0.8144	0.6229
	**Non-BSW (150)**	0.9552	0.9394	0.9138	0.8819	0.8405	0.7677	0.5442

	**Non-BSW (75)**	0.9526	0.9342	0.9042	0.8676	0.8218	0.7443	0.5291
	**Non-BSW (30)**	0.9444	0.9208	0.8818	0.8361	0.7837	0.7061	0.5097
**Recall**	**BSW (150)**	**0.9700**	**0.9565**	**0.9380**	**0.9144**	**0.8810**	**0.8167**	**0.6423**
	**BSW (75)**	0.9677	0.9515	0.9293	0.9008	0.8607	0.7858	0.6064
	**BSW (30)**	0.9591	0.9367	0.9059	0.8686	0.8189	0.7394	0.5679
	**Multibreed (50%)**	0.9665	0.9528	0.9338	0.9092	0.8736	0.8018	0.6170
	**Multibreed (25%)**	0.9650	0.9500	0.9293	0.9034	0.8665	0.7921	0.5975
	**Multibreed (10%)**	0.9628	0.9459	0.9222	0.8934	0.8535	0.7756	0.5702
	**Non-BSW (150)**	0.9523	0.9317	0.8993	0.8596	0.8100	0.7243	0.4961
	**Non-BSW (75)**	0.9457	0.9222	0.8849	0.8394	0.7835	0.6939	0.4760
	**Non-BSW (30)**	0.9282	0.8995	0.8526	0.7974	0.7350	0.6475	0.4535
**Precision**	**BSW (150)**	0.9626	**0.9586**	**0.9512**	**0.9398**	**0.9218**	**0.8839**	**0.7534**
	**BSW (75)**	**0.9632**	0.9571	0.9472	0.9331	0.9118	0.8672	0.7262
	**BSW (30)**	0.9625	0.9507	0.9340	0.9138	0.8863	0.8373	0.6862
	**Multibreed (50%)**	0.9620	0.9580	0.9507	0.9392	0.9208	0.8788	0.7383
	**Multibreed (25%)**	0.9609	0.9559	0.9473	0.9346	0.9152	0.8709	0.7171
	**Multibreed (10%)**	0.9599	0.9530	0.9419	0.9269	0.9051	0.8572	0.6864
	**Non-BSW (150)**	0.9583	0.9473	0.9287	0.9054	0.8746	0.8167	0.6027
	**Non-BSW (75)**	0.9597	0.9465	0.9245	0.8978	0.8641	0.8026	0.5956
	**Non-BSW (30)**	0.9611	0.9431	0.9133	0.8788	0.8392	0.7763	0.5818

Numbers in pure panels indicate the number of samples. Percentages in multibreed panels indicate the proportion of BSW samples. The top values for each metrics and coverage are highlighted.

***Table S6**. Compute resources used by DeepVariant (DV) and GATK to pre-process aligned BAM files, call variants per sample (gVCF stage), and jointly genotype and filter variants (pVCF stage).*

Variant caller	Pre-processing (h)	gVCF stage (h)	pVCF stage (h)	Peak memory (GB)
DV	-	577	3	81
GATK	820	999	233	49

Times are listed as CPU hours, and peak memory usage across all stages is given in gigabytes. DV does not require pre-processing. Jobs were submitted to nodes with different CPUs and non-exclusive use, and so these figures do not represent precise benchmarking. However, any node/usage variability is minor compared to the differences in total CPU hours used between DV and GATK.
